# No effect of monthly supplementation with 12000 IU, 24000 IU or 48000 IU vitamin D3 for one year on muscle function: The vitamin D in older people study

**DOI:** 10.1016/j.jsbmb.2018.12.008

**Published:** 2019-06

**Authors:** R.M.T.K. Ranathunga, T.R. Hill, J.C. Mathers, R.M. Francis, A. Prentice, I. Schoenmakers, T.J. Aspray, for the Vitamin D in Older People Study group

**Affiliations:** a Human Nutrition Research Centre, Institute of Cellular Medicine, Faculty of Medical Sciences, Newcastle University, Framlington Place, Newcastle upon Tyne, UK, NE2 4HH; b Institute of Cellular Medicine, Faculty of Medical Sciences, Newcastle University, Framlington Place, Newcastle upon Tyne, UK, NE2 4HH; c MRC Elsie Widdowson Laboratory, Cambridge, UK, CB1 9NL; d Norwich Medical School, University of East Anglia, Norwich, UK, NR4 7TJ; e NIHR Newcastle Biomedical Research Centre, Campus for Ageing and Vitality, Newcastle upon Tyne, UK, NE4 5PL; f Department of Applied Nutrition, Wayamba University of Sri Lanka, Faculty of Livestock, Fisheries and Nutrition, Makandura, Gonawila, 60170, Sri Lanka

**Keywords:** Older adults, Vitamin D supplementation, Muscle function, Grip strength, Timed-up and go test

## Abstract

Vitamin D plays a role in muscle function through genomic and non-genomic processes. The objective of this RCT was to determine the effect of monthly supplemental vitamin D_3_ onmuscle function in 70+ years old adults. Participants (n = 379) were randomized to receive, 12,000 IU, 24,000 IU or 48,000 IU of vitamin D_3_ monthly for 12 months. Standardized Hand Grip Strength (GS) and Timed-Up and Go (TUG) were measured before and after vitamin D_3_ supplementation. Fasting total plasma 25 hydroxyvitamin D (25OHD) and Parathyroid Hormone (PTH) concentrations were measured by Liquid Chromatography Tandem Mass Spectrometry (LC-MSMS) and immunoassay, respectively. Baseline plasma 25OHD concentrations were 41.3 (SD 19.9), 39.5 (SD 20.6), 38.9 (SD 19.7) nmol/L; GS values were 28.5 (SD 13.4), 28.8 (SD 13.0) and 28.1 (SD 12.1) kg and TUG test values were 10.8 (SD 2.5), 11.6 (SD 2.9) and 11.9 (SD 3.6) s for the 12,000 IU, 24,000 IU and 48,000 IU dose groups, respectively. Baseline plasma 25OHD concentration < 25 nmol/L was associated with lower GS (*P* = 0.003). Post-interventional plasma 25OHD concentrations increased to 55.9 (SD 15.6), 64.6 (SD15.3) and 79.0 (SD 15.1) nmol/L in the 12,000 IU, 24,000 IU and 48,000 IU dose groups, respectively and there was a significant dose-related response in post-interventional plasma 25OHD concentration (p<0.0001). Post-interventional GS values were 24.1 (SD 10.1), 26.2 (SD10.6) and 25.7 (SD 9.4) kg and TUG test values were 11.5 (SD 2.6), 12.0 (SD 3.7) and 11.9 (SD 3.2) s for 12,000 IU, 24,000 IU and 48,000 IU dose groups, respectively. The change (Δ) in GS and TUG from pre to post-intervention was not different between treatment groups before and after the adjustment for confounders, suggesting no effect of the intervention. Plasma 25OHD concentration was not associated with GS and TUG test after supplementation. In conclusion, plasma 25OHD concentration < 25 nmol/L was associated with lower GS at baseline. However, monthly vitamin D_3_ supplementation with 12,000 IU, 24,000 IU and 48,000 IU, for 12 months had no effect on muscle function in older adults aged 70+ years.

Trial Registration : EudraCT 2011-004890-10 and ISRCTN35648481.

## Highlights

• The effect of vitamin D on muscle strength, assessed by Grip Strength (GS) and function by Timed-up and Go test (TUG), were assessed in older people.
• We found that a plasma 25-hydroxyvitamin D <25 nmol/L at baseline was associated with lower GS.
• There was no dose effect on GS or TUG, comparing 12000IU, 24000IU or 48000IU of vitamin D per month for one year in people aged 70+ years.
• However, in the absence of a placebo group, we have cannot exclude a possible benefit of all three doses.

## 1 Introduction

Loss of muscle mass and decreased muscle strength are features of ageing, with an annual loss of muscle mass of 0.5–1.0% per year after 70 years of age [1] and a 10–15% decline in muscle strength per decade in older people aged 70–79 years [2]. Decreased muscle mass and strength can result in sarcopenia, which is associated with poorer quality of life, loss of independence and increased health care costs [3]. Assessment of Hand Grip Strength (GS) and Timed-Up and Go (TUG) are the widely used methods to test the muscle strength and identify the presence of sarcopenia [4].

Links between vitamin D status and muscle function have been reported based on mechanistic *in vitro* studies [5], human observational [[6], [7], [8], [9], [10], [11]], longitudinal [12] and intervention studies [13,14]. Some observational [6,9,13] and longitudinal [12] studies have reported positive associations between serum 25-hydroxyvitamin D (25OHD) concentration and muscle function in older adults, whereas other studies did not find an association [11]. These conflicting findings may be due to the differences in the characteristics of the population and differences in the vitamin D status of the participants. Current evidence suggests that vitamin D status is associated with reduced muscle strength, function and physical performance in older adults (over 60 years of age) only when serum 25OHD concentration falls below 50 nmol/L [15]. The scientific advisory committee on nutrition (SACN) recommended that serum 25OHD concentration should be at least 25 nmol/L all year round for optimal bone and muscle health [16].

The findings from vitamin D intervention studies are inconsistent, reflecting the variation in characteristics of the study population (e.g. age, gender, baseline vitamin D status), study design and nature of the intervention (route, dose, frequency and form of vitamin D supplementation). Some studies show the positive effect of vitamin D supplementation on muscle function only in older adults whose baseline serum 25OHD concentrations <30 nmol/L [17] or <50 nmol/L [15].

Since the plasma concentration and vitamin D supply required for optimal muscle function in older adults are not well understood, we undertook a secondary analysis of a 1-year dose-ranging randomised vitamin D_3_ supplementation trial, to evaluate its effects on muscle function [18].

## 2 Materials and methods

Vitamin D in older people (VDOP) study was a randomized double-blind interventional trial in 379 male and female older adults aged 70 years or older, living in the North-East of England (55 °N), which recruited from November 2012 and May 2013. The primary aim of this study was to assess the effect of monthly dose of 12,000 IU, 24,000 IU or 48,000 IU (equivalent to 400 IU, 800 IU or 1600 IU per day) vitamin D_3_ (Vigantol, Merck Sereno GmbH, Darmstadt Germany) on bone mineral density [18].

Potential participants were identified through screening of the electronic records of 25 GP practices. Exclusion criteria comprised: taking vitamin D supplements at a dose greater than 400 IU/day or calcium at a dose greater than 500 mg/day, a fragility fracture within the previous 6 months, treatment with an anti-resorptive or anabolic treatment for osteoporosis in the previous three years, a history of renal stones, previous hip replacement or primary hyperparathyroidism, hypercalcaemia (albumin adjusted plasma calcium >2.60 mmol/L), hypocalcaemia (albumin adjusted plasma calcium < 2.15 mmol/L) or an estimated glomerular filtration rate (eGFR) less than 30 ml/min/1.73 m^2^). Ethical permission was given by the Tyne and Wear Research Ethics Committee (REC,12/NE/0050). All participants provided written informed consent. The sponsor, Newcastle upon Tyne NHS Foundation Trust, provided the Research and Development approval for the study (Trial registration: EudraCT 2011 – 004890-10 and ISRCTN 35648481). Further details about participant recruitment are published elsewhere [19].

### 2.1 Intervention and study visits

Participants were randomized to receive one of three doses of vitamin D_3_, 12,000 IU, 24,000 IU or 48,000 IU, monthly for one year. Both participants and investigators were blinded to the treatment received. Study visits took place at baseline and thereafter at 3-monthly intervals (5 in total). Participants were provided with 3-monthly supplies of vitamin D_3_ at each study visit.

### 2.2 Outcome measures

GS (kg) in both the right and left hand was measured using a Jamar hand-grip dynamometer (Jamar, Bollington, USA). Three measurements were taken, and the mean value was used for analysis. The TUG test was performed once and recorded as the time taken in seconds (s) to stand from a sitting position in an arm chair and walk 3 m distance [20]. GS and TUG were measured before and after 1 year of supplementation. Anthropometry, including, height, weight, Fat Mass (FM) and Fat-Free Mass (FFM) were measured at three-month intervals. Height was measured using a stadiometer and weight, FM and FFM were measured using a bioelectrical impedance analyser (Tanita Crop, Tokyo, Japan). Habitual dietary vitamin D intake was assessed using a food frequency questionnaire (FFQ) at the screening visit, the 3^rd^ and 5^th^ visits. The vitamin D intake was calculated as the mean value of FFQ data of screening visit, the 3^rd^ and the 5^th^ visits.

### 2.3 Biochemical analysis

Overnight fasting venous blood samples were collected from participants at each visit. The 25OHD_2_ and 25OHD_3_ concentrations in plasma were measured by Liquid Chromatography Tandem Mass Spectrometry (LC-MSMS), before and after the intervention. Total 25OHD concentration was calculated by summing 25OHD_2_ and 25OHD_3_ values. EDTA plasma was used for the analysis of PTH by immunoassay (Immulite, Siemens Healthcare Diagnostics Ltd, Camberley, UK). Quality assurance of 25OHD and PTH assay were performed as the part of vitamin D Quality Assessment Scheme (http://www.deqas.org/) and the National External Quality Assessment Scheme (https://ukneqas.org.uk/). Inter assay variations were < 10% and < 7% for 25OHD_2_ and 25OHD_3_, respectively.

### 2.4 Data and statistical analysis

Baseline data were available for 379 older adults, while 343 older adults completed the intervention study. Thus, the total sample of 379 was used for the baseline data analysis, while the data from 343 older adults were used to investigate the intervention effects after 12 months. Older adults were sub-divided into two groups based on baseline plasma 25OHD concentration < 25 nmol/L, which is the cut-off of value of vitamin D used in the UK to indicate an increased risk of deficiency [16] and plasma 25OHD concentration < 50 nmol/L, which is the cut-off for vitamin D inadequacy used in North America [21], both of which have recently been incorporated in to the National Osteoporosis guidelines in the UK [22]. Statistical analysis of the data was conducted using SPSS for Windows version 13.0. Kolmogorov-Smirnov test was used to evaluate the distribution of the variables and those that were not normally distributed were log transformed prior to the analysis and were near normally distributed after the conversion. Primary outcomes for the study were GS and TUG in response to supplementation with 12,000 IU, 24,000 IU and 48,000 IU vitamin D_3_ per month.

Baseline 25OHD, baseline muscle function variables, age, weight, height, FM, FFM and vitamin D intake were predetermined as potential confounders. Multinomial logistic regression analysis was used to investigate the association between muscle function at baseline and plasma 25OHD concentration (based on whether plasma level was above or below two cut-offs: 25 nmol/L and 50 nmol/L), adjusting for confounders. The ANOVA test was used to test the effect of vitamin D supplementation on muscle function, plasma 25OHD and PTH concentrations. ANCOVA was used to test for the effect of the treatment on post-intervention variables after controlling for potential confounders (age, weight, height, Fat Mass (FM), Fat Free Mass (FFM) and vitamin D intake). The Bonferroni test was used for *post hoc* comparisons. Multiple linear regression was used to test potential effect of plasma 25OHD concentration on muscle function after supplementation. A *P* value <0.05 was considered as significant.

## 3 Results

Table 1 presents the participants’ characteristics at baseline, stratified by vitamin D_3_ supplementation dose. Baseline values for the main outcome measures, GS, TUG and plasma 25OHD concentration were similar across the intervention groups as were mean values for the main confounders including weight, height, BMI and age indicating that randomization was successful. The initial characteristics of the baseline sample (379 participants) and the sample of older adults who completed the intervention study (343 participants) were similar (data are not shown).

**Table 1:** Participants’ characteristics at baseline by the dose of vitamin D supplementation.

Characteristics	12,000 IU			24,000 IU			48,000 IU		
	n (%)	Mean	SD	n (%)	Mean	SD	n (%)	Mean	SD
Age (years)	126	74.6	4.0	125	75.0	4.2	128	75.4	4.4
Age (n, % >70 < 71.5)	33 (26.2)			31 (24.8)			33 (25.8)		
Age (n,% > 71.5 < 74)	36 (28.6)			30 (24.6)			28 (21.9)		
Age (n,% > 74 < 77)	29 (23.0)			33 (26.4)			29 (22.7)		
Age (n,% > 77)	28 (22.2)			31 (24.8)			38 (29.7)		
Gender (n, % males)	126 (54.8)			125 (52.8)			128 (49.2)		
Weight (kg)	126	73.9	11.8	125	77.1	14.0	128	76.1	14.2
Height (cm)	126	167.4	8.1	125	167.0	9.8	128	167.4	10.0
Waist (cm)	125	94.5	11.4	125	97.7	14.0	127	97.5	14.3
Hip (cm)	125	103.9	8.2	125	105.8	9.5	127	105.3	10.5
BMI^1^ (kgm^−2^)	126	26.3	3.6	124	27.5	4.1	127	27.2	4.0
<18.5	0 (0.0) 50 (41.0) 60 (32.3) 16 (33.2)			1 (0.8) 31 (25.4) 65 (34.9) 28 (33.0)			0 (0.0) 41 (33.6) 61(32.8) 26 (33.8)		
18.5 – 24.9									
25.0 – 29.9									
>30.0									
Body fat %	124	31.9	8.6	125	32.9	7.7	127	32.5	7.8
GS^2^ (kg)	126	28.5	13.4	124	28.8	13.0	127	28.1	12.1
TUG^3^ (s)	125	10.8	2.5	124	11.6	2.9	127	11.9	3.6
Plasma 25OHD^4^ (nmol/L)	126	41.3	19.9	124	39.5	20.6	128	38.9	19.7
PTH^5^ (Pg/ml)	126	48.6	25.7	123	47.4	23.3	128	49.9	21.3
Dietary vitamin D intake (μg/day)	119	3.6	2.0	121	3.6	2.5	123	4.0	3.0

1Body Mass Index 2 Grip Strength 3 Timed-Up and-Go4 25-hydroxy vitamin D 5Parathyroid Hormone.

Table 2 shows the multinomial logistic regression analysis of the relationships between baseline plasma 25OHD concentration and muscle function variables according to the cut-offs values of plasma 25OHD concentrations from SACN, 2016 (25 nmol/L) and North American Institute of Medicien (IOM), 2011 (50 nmol/L). After adjusting for age, body weight, height, FM, FFM and vitamin D intake, older adults who had plasma 25OHD concentration < 25 nmol/L at baseline were significantly (p = 0.003) less likely to have GS above the median compared with individuals with plasma 25OHD concentration >25 nmol/L. This relationship was evident for GS for both males (p = 0.015) and females (p = 0.050). When using the cut-off value of 50 nmol/L, there was no relationship between vitamin D status and either GS or TUG for all participants and for both gender groups.

**Table 2:** Multinomial logistic regression analysis1 of relationships between plasma 25OHD concentration, categorized according to SACN and IOM cut-offs, and muscle function at baseline.

	Total population (n = 379)			Males (n = 198)			Females (n = 181)		
Classification	OR	CI	P value*	OR	CI	*p* value**	OR	CI	*P* value**
**GS (kg)**									
25OHD < 25 nmol/L^2^	0.339	0.166 – 0.691	0.003	0.333	0.137 – 0.810	0.015	0.251	0.063 – 1.001	0.050
25OHD >25 nmol/L	**Reference**			**Reference**			**Reference**		
25OHD < 50 nmol/L^3^	0.990	0.510 – 1.922	0.976	0.588	0.216 – 1.443	0.229	1.870	0.520 – 6.719	0.338
25OHD >50 nmol/L	**Reference**			**Reference**			**Reference**		
**TUG (s)**									
25OHD < 25 nmol/L	0.645	0.388 – 1.070	0.090	0.501	0.229 – 1.093	0.084	0.720	0.358 – 1.446	0.355
25OHD >25 nmol/L	**Reference**			**Reference**			**Reference**		
25OHD < 50 nmol/L	0.697	0.424 – 1.147	0.155	0.775	0.386 – 1.543	0.468	0.584	0.273 – 1.250	0.166
25OHD > 50 nmol/L	**Reference**			**Reference**			**Reference**		

1To be in the category of higher muscle function based on dichotomisation at the median value ^2^ SACN cut-off ^3^IOM cut-off.

* Adjusted for gender, age, body weight, height, fat mass (FM), fat free mass (FFM) and dietary vitamin D intake.

** Adjusted for age, body weight, height, FM, FFM and dietary vitamin D intake.

After one year of vitamin D supplementation, there were no differences between treatment doses for post-intervention GS or TUG. In addition, there were no significant changes in GS and TUG from baseline between intervention arms, with and without adjustment for baseline values, age, gender, weight, height, FM, FFM and vitamin D intake. Further, subgroup analysis of those with a baseline 25OHD concentration < 50 and <25 nmol/L, did not show any significant differences between intervention arms in post-intervention GS and TUG, or for change in GS and TUG. As expected, there were significant differences between treatment arms in post-interventional plasma 25OHD and change in 25OHD concentration. This relation was the same for the sub-group analysis in those with baseline plasma 25OHD concentration < 50 nmol/L and < 25 nmol/L. After the supplementation, the mean change in plasma 25OHD concentration was 14.3 (SD 12.6), 25.3 (SD 18.0) and 40.9 (SD 19.8) nmol/L for the 12,000 IU, 24,000 IU and 48,000 IU dose group, respectively. There was no significant difference between the groups in unadjusted PTH post-intervention, although the decrease in PTH was significant larger at the highest dose after correction for confounders. In subgroup analyses, change in PTH was significantly different between intervention arms before and after adjusting for confounders comparing those with baseline 25OHD concentrations equal to or above with those below 50 nmol/L at baseline but this was not the case for the 25 nmol/L cut point (Table 3).

**Table 3:** Effect of vitamin D supplementation on post-interventional and change (Δ) in muscle function variables, plasma 25OHD concentration and PTH concentration by the dose of vitamin D supplementation.

Parameters	12,000 IU/month	24,000 IU/month	48,000 IU/month	*p*1	*p*2
Total sample (n = 343)					
**Plasma 25OHD**^3^**(nmol/L)**	**(n = 113)**	**(n = 114)**	**(n = 116)**		
Pre-intervention	41.2 (20.3)	39.4 (20.8)	38.5 (19.4)	0.495	
Post-intervention	55.9 (15.6)	64.6 (15.3)	79.0 (15.1)*	<0.0001	<0.0001
Change (Δ) in 25OHD	14.3 (12.6)	25.3 (18.0)	40.9 (19.8)*	<0.0001	<0.0001
**PTH^4^ (pg/mL)**					
Pre-intervention	46.8 (23.5)	47.1 (23.9)	50.6 (21.6)	0.443	
Post-intervention	44.0 (21.3)	44.6 (24.5)	40.1 (18.4)	0.244	0.016
Change (Δ) in PTH	−2.9 (18.4)	−3.1 (18.2)	−10.6 (15.4)*	<0.0001	0.001
**GS^5^ (kg)**					
Pre-intervention	27.5 (12.7)	29.4 (13.2)	28.1 (12.2)	0.641	
Post-intervention	24.7 (10.1)	26.2 (10.6)	25.7 (9.4)	0.692	0.449
Change (Δ) in GS	−2.8 (11.6)	−3.2 (8.1)	−2.4 (7.7)	0.820	0.426
**TUG^6^ (s)**					
Pre-intervention	10.9 (2.5)	11.5 (2.9)	11.8 (3.5)	0.187	
Post-intervention	11.5 (2.6)	12.0 (3.7)	11.9 (3.2)	0.437	0.713
Change (Δ) in TUG	0.56 (2.32)	0.46 (2.77)	0.15 (2.5)	0.773	0.680
**Older adults with baseline 25OHD < 50 nmol/L (n = 242)**					
**Plasma 25OHD (nmol/L)**	**(n = 75)**	**(n = 83)**	**(n = 84)**		
Pre-intervention	30.2 (10.9)	29.1 (10.3)	29.6 (10.6)	0.862	
Post-intervention	49.7 (11.8)	60.5 (14.8)	76.8 (14.3)*	<0.0001	<0.0001
Change (Δ) in 25OHD	19.3 (10.0)	31.3 (15.0)	47.4 (15.3)*	<0.0001	<0.0001
**PTH (pg/mL)**					
Pre-intervention	49.9 (24.9)	52.9 (24.4)	54.2 (22.0)	0.210	
Post-intervention	45.7 (21.5)	48.9 (24.5)	41.0 (19.7)	0.137	0.101
Change (Δ) in PTH	−4.2 (21.1)	−4.0 (19.5)	−13.4 (15.3)*	0.001	0.002
**GS (kg)**					
Pre-intervention	26.5 (11.8)	29.1 (13.6)	28.3 (12.8)	0.457	
Post-intervention	23.8 (10.3)	25.5 (10.3)	25.1 (9.3)	0.801	0.403
Change (Δ) in GS	−2.7 (12.5)	−3.6 (8.5)	−3.1 (8.4)	0.486	0.889
**TUG (s)**					
Pre-intervention	10.9 (2.3)	11.8 (2.9)	11.8 (3.2)	0.094	
Post-intervention	11.7 (2.8)	12.3 (3.9)	12.0 (3.5)	0.560	0.630
Change (Δ) in TUG	0.74 (2.4)	0.45 (2.8)	0.29 (2.3)	0.512	0.823
**Older adults with baseline 25OHD < 25 nmol/L (n = 93)**					
**Plasma 25OHD (nmol/L)**	**(n = 31)**	**(n = 30)**	**(n = 32)**		
Pre-intervention	19.3 (4.1)	18.2 (4.5)	18.8 (3.9)	0.582	
Post-intervention	41.9 (10.7)	55.5 (14.7)	73.7 (12.8)*	<0.0001	<0.0001
Change (Δ) in 25OHD	22.6 (9.3)	37.3 (14.0)	54.9 (12.7)*	<0.0001	<0.0001
**PTH (pg/mL)**					
Pre-intervention	61.9 (27.8)	59.5 (19.3)	58.8 (23.2)	0.930	
Post-intervention	50.4 (20.6)	47.7 (16.6)	41.5 (24.1)	0.102	0.143
Change (Δ) in PTH	−12.2 (25.9)	−11.9 (17.4)	−17.4 (14.7)	0.470	0.563
**GS (kg)**					
Pre-intervention	25.6 (13.8)	24.1 (9.1)	25.8 (14.2)	0.895	
Post-intervention	22.3 (11.2)	22.5 (9.0)	22.8 (7.9)	0.903	0.860
Change (Δ) in GS	−3.3 (16.1)	−1.6 (4.8)	−3.0 (9.1)	0.814	0.714
**TUG (s)**					
Pre-intervention	11.0 (2.4)	12.8 (3.7)	12.5 (4.1)	0.938	
Post-intervention	11.9 (2.8)	13.7 (4.1)	12.9 (4.1)	0.798	0.379
Change (Δ) in TUG	0.91 (2.6)	0.88 (2.7)	0.38 (2.5)	0.806	0.823

1One-way ANOVA followed by Bonferroni test.

2ANCOVA controlled for baseline values of the variables, age, gender, weight, height, Fat Mass (FM), Fat Free Mass (FFM) and vitamin D intake.

325-hydroxyvitamin D 4 Parathyroid Hormone5Grip Strength 6Timed-Up and Go test.

* Significantly different from 12,000 IU and 24,000 IU groups.

After supplementation, plasma 25OHD concentration was not significantly associated with either GS or TUG. Significant determinants for GS after supplementation were height (p < 0.0001), gender (p=<0.0001), age (p = 0.002), concurrent body weight (p = 0.040) and FM (p = 0.040). Similarly, the determinants of TUG were age (p < 0.0001), height (p < 0.0001), fat mass (p=<0.0001), gender (p = 0.018) and vitamin D intake (p = 0.019) (data are not shown).

## 4 Discussion

### 4.1 Main findings

This double-blind, randomized controlled study found that monthly vitamin D_3_ supplementation with 12,000 IU, 24,000 IU and 48,000 IU (which corresponds to 400, 600 and 1200 μg of dietary vitamin D per day) for one year produced significant dose-related increases in plasma 25OHD concentration but had no effect on muscle function in older adults. However, at baseline, there was an association between plasma 25OHD concentration and GS, with significantly lower GS for those with baseline plasma 25OHD concentration <25 nmol/L in both males and females. After supplementation, there were no associations between plasma 25OHD concentration and muscle function. To our knowledge, this is the first dose-ranging RCT conducted in the UK, with a large number of free-living older adults, evaluating the effect of vitamin D supplementation on muscle function.

### 4.2 Comparison with other studies

In line with our findings, previous RCTs reported that vitamin D supplementation had no beneficial effect on muscle function in older adults, irrespective of the dose of vitamin D supplements given. A recent study of female adults of long-term care residence aged 65+ years, supplemented with the oral dose of 800 IU vitamin D_3_ daily for 24 months, reported no effect of the supplementation on muscle function measured by gait speed and physical performance test [23]. Another study that conducted recruiting home-dwelling men and women aged > 70 years who were randomized to receive one of the oral doses of 24,000 IU, 60,000 IU or 24,000 IU vitamin D_3_ with 300 μg of calcifediol monthly for 12 months, reported no improvement in lower extremity function measured by short physical performance battery [24]. Hansen et al., 2015 reported that among postmenopausal women aged 75 years or younger with baseline 25OHD concentration 14–27 ng/mL, (˜35–67.5 nmol/L) and supplemented with an oral dose of 800 IU or 50,000 IU vitamin D_3_ twice monthly for one year had no effect on muscle function, assessed by the ‘five sit-stand’ test and TUG test [25]. Further, a recent systematic review of community-dwelling older adults aged 65+ years showed that bolus injection or oral vitamin D supplementation of dose ranging from 1000 IU – 600,000 IU, given daily or weekly for duration ranging from 16 weeks to 20 months, had no effect on GS and TUG test [26].

In contrast, some RCTs have shown improvements in muscle function following vitamin D supplementation in older adults. A study conducted among residents of nursing homes of average age of 89 years, who had been randomized to receive one of four oral dose of vitamin D_3_ supplements (200 IU, 400 IU, 600 IU and 800 IU) or placebo daily for 5 months, showed that those receiving the highest dose had the lowest number of falls compared to the other groups [27]. Positive effects of oral vitamin D_3_ supplements on GS and chair rise test were reported in a study of postmenopausal women aged 50–65 years who received 1000 IU of oral vitamin D_3_ daily for 9 months [28]. A study of ambulatory older adults with the history of falls and serum 25OHD < 12 μg/L (˜ 30 nmol/L) who received a single intramuscular injection of 60,000 IU of ergocalciferol reported a beneficial effect on functional performance, reaction time and balance but not on muscle strength [29]. Another RCT of ambulatory older adults live in a nursing home with a serum 25OHD concentration < 30 ng/mL (˜ 75 nmol/L), randomized to receive the oral or intramuscular injection of 600000 IU of cholecalciferol for 12 weeks, demonstrated an improvement in muscle strength assessed using quadriceps and physical performance battery [30]. According to Zhu et al., 2010, among community-dwelling older adults aged 70–90 years with serum 25OHD concentration < 24 ng/ml (˜ 60 nmol/L) supplemented with 1000 IU of vitamin D_2_ daily for 1 year, improved TUG test only among the older adults who were the slowest and weakest at the baseline [31]. Similarly, a systematic review with a meta-analysis reported that vitamin D supplementation had a positive effect on muscle function in older adults whose baseline serum 25OHD concentration was < 25 nmol/L [32]. These inconsistent findings are likely attributed to differences in study design including cohort characteristics, duration, dosage, formulations, route of vitamin D supplementation and the functional outcomes measured.

To support our finding of an association between GS and plasma 25OHD concentration below 25 nmol/L at baseline, Wu et al., 2017, reported that the serum 25OHD concentration of 29–33 nmol/L may optimise musculoskeletal health in middle-aged women (36–57 years) [33]. Similar to our study Grimaldi et al., 2013, also reported a positive association between serum 25OHD concentration and GS, but not with other tests of muscle function and suggested that this might be related to anatomical site differences in the androgenic effect of vitamin D or to differences in vitamin D receptor expression between upper and lower body muscle and consequently muscle function [34].

GS loss in our study was much higher than the reported values in previous studies. The annual loss of GS among the older people aged 65–75 years reported in previous studies ranged from 0.3 to 1.3 kg [[35], [36], [37]]. Though the GS is the standard method to assess sarcopenia, differences in the equipment and methods used in various studies may have caused variation in the measurements, making it difficult to compare between studies [38].

In this study we found that plasma 25OHD concentration < 25 nmol/L was associated with a lower GS. This finding supports the recommendation of SACN, UK that for the protection of musculoskeletal health, serum 25OHD concentration should not fall below 25 nmol/L throughout the year [16]. The US Institute of Medicine (IOM) defines the desired range of 25OHD as 30–50 nmol/L and ESFA (European Food Safety Authority [39];) advises a target value of 50 nmol/L for the general population. The US Endocrine Society advises a target range of > 50 nmol/L for patient management who are at risk of vitamin D deficiency [40]. In addition, Kotlarczyk et al., 2017 suggested that at least a concentration between 30–40 ng/ml (˜75–100 nmol/L) is required for older adults for optimum muscle function [23]. With regards to vitamin D supplementation, the American Geriatrics Society [41] and the Endocrine Society [40] recommend vitamin D supplementation of 600–1000 IU/day in older adults who are at risk of falling. A systematic review of vitamin D supplementation trials also reported a daily dose of 700–1000 IU for physical performance and to prevent falls [42]. SACN, 2016 suggests that for the adults > 50 years, the beneficial effect of vitamin D supplementation on muscle strength and function can be seen among the adults at the mean baseline serum 25OHD concentrations ranging between 25 and 66 nmol/L. In our study, none of the intervention groups reached a mean post-intervention plasma 25OHD concentration above this range. Accordingly, it can be speculated that the dosages used in this study may not have been high enough to reduce the negative effects of the ageing process. The absence of a detectable effect of supplementation in this study may also be attributed to the fact that only 30% of our participants had a 25OHD < 25 nmol/l at baseline. However, as a result of lack of a placebo group we did not have data on “natural decline” of muscle function of this group, thus we could not compare the effect of vitamin D supplementation with that in non-supplemented individuals.

For tissues other than the kidney, total 25OHD may not fully reflect its availability for local hydroxylation into 1,25(OH)_2_D, which is the active metabolite of vitamin D. Although 1,25(OH)_2_D is responsible for the biological action of vitamin D, its systemic concentration does not reflect function at the target tissue level [43,44]. Many vitamin D target tissues, including the muscle tissue, are known to express the 1,25(OH)_2_D-producing enzyme CYP27A1 for auto- and paracrine functions. Some reports suggest that muscle tissue may be capable of internalising vitamin D binding protein bound 25OHD, although it remains to be determined whether this is a significant route of cellular supply of 25OHD [8]. To date, no data are available to identify whether free 25OHD provides a better prediction of muscle function compared to total 25OHD.

### 4.3 Limitations, strengths and future studies

We used the lowest intervention dose (which corresponds to the current UK dietary recommendation) as the reference group but did not include a placebo group in our study design as directed by the approving authorities. As a result, we could not establish the effect of three doses of vitamin D supplements compared to a placebo group. Further, we only measured plasma 25OHD concentration at baseline and after 1-year supplementation. These samples were collected early winter to late spring, during which vitamin D status is lower than the year-round average in non-supplemented individuals. Therefore, the vitamin D status of individuals at baseline and post-intervention may have been misclassified as it may not have fully reflected the trajectory of vitamin D status throughout the year. Further, this population was not selected randomly from the community. They were invited on the basis of screening of pre-specified criteria in their electronic health record. Also, there may have been self-selection bias as those that expressed an interest to participate in the study may have been more health conscious. In addition, the response to vitamin D supplementation and status may have been influenced by factors not measured in this study, such as the distribution of type I and type II of muscle fibres, genetic factors, habitual physical activity or exercise habits and hormonal factors.

This study had several strengths. Its large sample size and the number of available measurements that could potentially influence muscle force, i.e. those related to body composition and size. The use of three different vitamin D doses corresponding to the UK Recommended Nutrient Intake (RNI) [16], the US Recommended Dietary Allowances (RDA) [21] and the value below the Tolerable Upper Intake Level (TUIL) was a strength of this study. We considered the effect of vitamin D supplementation, without an exercise intervention, on muscle. Few trials have looked at the potential interaction between exercise and vitamin D, although a recent systematic review presented evidence of an additive effect of resistance exercise and vitamin D3 supplementation for the improvement of muscle strength in older adults, and we would support suggestions that this is an important area for future research [45].

## 5 Conclusions

At baseline, plasma 25OHD was associated with GS in both male and females, but only below the cut-off level of 25 nmol/L. Vitamin D supplementation significantly increased the plasma 25OHD concentration of older adults in all doses of supplementation. Vitamin D supplementation with 12,000 IU, 24,000 IU and 48,000 IU for 12 months had no effect on muscle function in adults older than 70 years.

## Funding and role of funders

This study was funded by Arthritis Research UK. Clinical Studies Grant Number 19544 and was supported by MRC programme number U105960371.

## Role of funders

The study design was internationally peer-reviewed for the Arthritis Research UK as part of the funding decision process. Reviewers’ recommendations were taken into account in designing the trial. Arthritis Research UK were not involved in the analyses or in the decision to publish these results from the trial.

## Role of sponsors

The sponsor was responsible for the conduct of the trial according to guidelines declared by GCP, GRP, the Data Protection Act and the Declaration of Helsinki. The sponsor was also responsible for Pharmacovigilance. These responsibilities were delegated to the Principal Investigator Dr T. Aspray and Newcastle CTU. The sponsor did not play any role in design, execution, analysis or interpretation of the data,

## Conflict of interests

Authors declare that they have no competing interests.

## References

[1] Mitchell W.K., Williams J., Atherton P., Larvin M., Lund J., Narici M. (2012). Sarcopenia, dynapenia, and the impact of advancing age on human skeletal muscle size and strength; a quantitative review. Front. Physiol..

[2] Goodpaster B.H., Park S.W., Harris T.B. (2006). The loss of skeletal muscle strength mass and quality in older adults: the health, aging and body composition study. J. Gerontol. Series A Biol. Sci. Med..

[3] Yu S., Umapathysivam K., Visvanathan R. (2014). Sarcopenia in older people’. Int. J. Evid. Based Health Care.

[4] Dodds R.M., Syddall H.E., Cooper R., Benzeval M., Deary I.J., Dennison E.M., Der G., Gale C.R., Inskip H.M., Jagger C., Kirkwood T.B., Lawlor D.A., Robinson S.M., Starr J.M., Steptoe A., Tilling K., Kuh D., Cooper C., Sayer A.A. (2014). Grip strength across the life course: normative data from twelve British studies. PLoS One.

[5] Bischoff-Ferrari H.A., Borchers M., Gudat F., Duermueller U., Theiler R., Stahelin H.B., Dick W. (2001). In situ detection of 1,25dihydroxyvitamin D3 receptor in human skeletal muscle tissues. J. Histochem. Cytochem..

[6] Toffanello E.D., Perissinotto E., Sergi G., Zambon S., Musacchio E., Maggi S., Coin A., Sartori L., Corti M.C., Baggio G., Crepaldi G., Manzato E. (2012). Vitamin D and physical performance in elderly subjects: the Pro.V.A study. PLoS One.

[7] Ceglia L., Harris S.S. (2013). Vitamin D and its role in skeletal muscle. Calcif. Tissue Int..

[8] Girgis C.M., Clifton-Bligh R.J., Hamrick M.W., Holick M.F., Gunton J.E. (2013). The roles of vitamin D in skeletal muscle form, function, and metabolism. Endocr. Rev..

[9] Tieland M., Brouwer-Brolsma E.M., Nienaber-Rousseau C., van Loon L.J., De Groot L.C. (2013). Low vitamin D status is associated with reduced muscle mass and impaired physical performance in frail elderly people. Eur. J. Clin. Nutr..

[10] Brech G.C., Ciolac E.G., Peterson M.D., Greve J.M. (2017). Serum 25-hydroxyvitamin D levels are associated with functional capacity but not with postural balance in osteoporotic postmenopausal women. Clinics.

[11] Berners A.V., Cederholm T., Fielding R.A., Gustafsson T., Kirn D., Laussen J., Nydahl M., Travison T.G., Feid K., Koochek A. (2018). Physical performence and serum 25(OH)D status in community dwelling old mobility limited adults: a cross sectional study. J. Nutr. Healthy Aging.

[12] Granic A., Hill T.R., Davies K., Jagger C., Adamson A., Siervo M., Kirkwood T.B., Mathers J.C., Sayer A.A. (2017). Vitamin D status, muscle strength and physical performance decline in very old adults: a prospective study. Nutrients.

[13] Muir S.W., Montero-Odasso M. (2011). Effect of vitamin D supplementation on muscle strength, gait and balance in older adults: a systematic review and meta-analysis. J. Am. Geriatr. Soc..

[14] Tomlinson P.B., Joseph C., Angioi M. (2015). Effects of vitamin D supplementation on upper and lower body muscle strength levels in healthy individuals. A systematic review with meta-analysis’. J. Sci. Med. Sport.

[15] Rejnmark L. (2011). Effects of vitamin D on muscle function and performance : a review of evidence from randomized controlled trials. Therapeut. Adv. Chronic Dis..

[16] Scientific Advisory Committee on Nutrition SACN (2016). Vitamin D and Health. https://www.gov.uk/government/publications/sacn-vitamin-d-and-health-report.

[17] Beaudart C., Buckinx F., Rabenda V., Gillain S., Cavalier E., Slomian J., Petermans J., Reginster J., Bruyère O. (2014). The effects of vitamin D on skeletal muscle strength, muscle mass, and muscle power: a systematic review and meta-analysis of randomized controlled trials. J. Clin. Endocrinol. Metab..

[18] Aspray T.J., Chadwick T., Francis R.M., McColll Stamp E., Prentice A., Wilamowitz-Moellendorff A., Schoenmakers I. (2018). Am. J. Clin. Nutr..

[19] Schoenmakers I., Francis M., McColl E., Chadwick T., Goldberg G., Harle C., Yarnall A.J.W., Parker J.P., Prentice A., Aspray T. (2013). Vitamin D supplementation in older people (VDOP): study protocol for a randomised controlled intervention trial with monthly oral dosing with 12,000 IU, 24,000 IU or 48,000 IU of vitamin D3. Trials.

[20] Weiss A., Herman T., Plotnik M., Brozgol M., Maidan I., Giladi N., Gurevich T., Hausdorff J.M. (2010). Can an accelerometer enhance the utility of the timed up & go Test when evaluating patients with Parkinson’s disease?. J. Med. Eng. Phys..

[21] Ross AC, Institute of Medicine (US) (2011). Committee to Review Dietary Reference Intakes for Vitamin D and Calcium. Dietary Reference Intakes : Calcium, Vitamin D.

[22] Francis R., Aspray T.J., Fraser W., MacDonald H., Patel S., Mavroeidi A., Schoenmakers I., Stone M. (2018). Vitamin D and Bone Health: A Practical Clinical Guideline for Patient Management.

[23] Kotlarczyk M.P., Perera S., Ferchak M.A., Nace D.A., Resnick N.M., SL G. (2017). ’Vitamin D deficiency is associated with functional decline and falls in frail elderly women despite supplementation. Osteoporos. Int..

[24] Bischoff-Ferrari H.A., Dawson-Hughes B., Orav E.J., Staehelin H.B., Meyer O.W., Theiler R., Dick W., Willett W.C., Egli A. (2016). Monthly high-dose vitamin D treatment for the prevention of functional decline a randomized clinical trial. JAMA Int. Med..

[25] Hansen K.E., Johnson R.E., Chambers K.R., Johnson M.G., Lemon C.C., Vo T.N., Marvdashti S. (2015). Treatment of vitamin D insufficiency in postmenopausal women: a randomized clinical trial. JAMA Int. Med..

[26] Rosendahl-Riise H., Spielau U., Ranhoff A.H., Gudbrandsen O.A., Dierkes J. (2017). Vitamin D supplementation and its influence on muscle strength and mobility in community-dwelling older persons: a systematic review and meta-analysis. J. Hum. Nutr. Diet..

[27] Broe K.E., Chen T.C., Weinberg J., Bischoff-Ferrari H.A., Holick M.F., Kiel D.P. (2007). A higher dose of vitamin d reduces the risk of falls in nursing home residents: a randomized, multiple-dose study. J. Am. Geriatr. Soc..

[28] Cangussu L.M., Nahas-Neto J., Orsatti L.C., Bueloni-Dias F.N., Nahas E.A.P. (2015). Effect of vitamin D supplementation alone on muscle function in postmenopausal women: a randomized, double-blind, placebo-controlled clinical trial. Osteoporos. Int..

[29] Dhesi J.K., Jackson S.H.D., Bearne L.M., Moniz C., Hurley M.V., Swift C.G., Allain T.J. (2004). Vitamin D supplementation improves neuromuscular function in older people who fall. Age Ageing.

[30] Tellioglu A., Basaran S., Guzel R., Seydaoglu G. (2012). Efficacy and safety of high dose intramuscular or oral cholecalciferol in vitamin D deficient/insufficient elderly. Maturitas.

[31] Zhu K., Austin N., Devine A., Bruce A., Prince L. (2010). A randomized control Trial of the effect of vitamin D on muscle strength and Mobility in older women with vitamin insufficiency. J. Am. Geriatr. Soc..

[32] Stockton K.A., Mengersen K., Paratz J.D., Kandiah D., Bennell K.L. (2011). Effect of vitamin D supplementation on muscle strength: a systematic review and meta-analysis. Osteoporos. Int..

[33] Wu F., Wills Laslett L.L., Oldenburg B., Seibel M.J., Jones G., Winzenberg T. (2017). Cut-points for associations between vitamin D status and multiple musculoskeletal outcomes in middle-aged women. Osteoporos. Int..

[34] Grimaldi A.S., Parker B.A., Capizzi J.A., Clarkson P.M., Pescatello L.S., White M.C., Thompson P.D. (2013). 25(OH) vitamin D is associated with greater muscle strength in healthy men and women. J. Med. Sci. Sports Exercercis..

[35] Turusheva A., Frolova E., Degryse J. (2017). Age-related normative values for handgrip strength and grip strength’s usefulness as a predictor of mortality and both cognitive and physical decline in older adults in northwest Russia. J. Musculoskelet. Neuronal Interact..

[36] Frederiksen H., Hjelmborg J., Mortensen J., McGue M., Vaupel J., Christensen K. (2006). Age trajectories of grip strength: cross-sectional and longitudinal data among 8,342 Danes aged 46 to 102. Ann. Epidemiol..

[37] Xue Q., Beamer B., Chaves P., Guralnik J., Fried L. (2010). Heterogeneity in rate of decline in grip, hip, and knee strength and the risk of all-cause mortality: the women’s health and aging study II. J. Am. Geriatr. Soc..

[38] Roberts H.C., Denison H.J., Martin H.J., Patel H.P., Syddall H., Cooper C., Sayer A.A. (2011). A review of the measurement of grip strength in clinical and epidemiological studies: towards a standardised approach. J. Age Ageing.

[39] EFSA NDA Panel on Dietetic Products, Nutrition and Allergies (2016). Scientiﬁc opinion on dietary reference values for vitamin D. EFSA J..

[40] Holick M.F., Binkley N.C., Bischoff-Ferrari H.A., Gordon C.M., Hanley D.A., Heaney R.P. (2011). Evaluation, treatment, and prevention of vitamin D deficiency :an Endocrine Society clinical practice guideline. J. Clin. Endocrinol. Metab..

[41] American Geriatrics Society Workgroup on Vitamin D (2014). Recommendations abstracted from the American Geriatrics Society consensus statement on vitamin D for prevention of falls and their consequences. J. Am. Geriatr. Soc..

[42] Bischoff-Ferrari H.A., Dawson-Hughes B., Staehelin H.B., Orav J.E., Stuck A.E., Theiler R., Wong J.B., Egli A., Kiel D.P., Henschkowski J. (2009). Fall prevention with supplemental and active forms of vitamin D: a meta-analysis of randomised controlled trials. Br. Med. J..

[43] Prentice A., Goldberg G.R., Schoenmakers I. (2008). Vitamin D across the lifecycle: physiology and biomarkers. Am. J. Clin. Nutr..

[44] Bikle D., Bouillon R., Thadhani R., Schoenmakers I. (2017). Vitamin D metabolites in captivity? Should we measure free or total 25(OH)D to assess vitamin D status?. J. Steroid Biochem. Mol. Biol..

[45] Antoniak A., Greig C. (2017). The effect of combined resistance exercise training and vitamin D3 supplementation on musculoskeletal health and function in older adults: a systematic review and meta-analysis. Br. Med. J..

